# Simultaneous Association of Pulmonary Tuberculosis and Kaposi's Sarcoma in an Immunocompetent Subject: A Case Report and Literature Review

**DOI:** 10.1155/2019/5453031

**Published:** 2019-10-27

**Authors:** Sofia Baina, Jihane Achrane, Jouda Benamor, Jamal Eddine Bourkadi

**Affiliations:** Pulmonology Department, Moulay Youssef Hospital, Faculty of Medicine and Pharmacy, Med V University, CHU, Rabat, Morocco

## Abstract

Kaposi's Sarcoma (KS) occurs as a pathological entity that may be classified into four different types: classic, endemic, epidemic, and iatrogenic. It can arise among HIV-positive subjects or within immunosuppression, yet exceptionally of tuberculous origin. We describe a new case report of an HIV-negative patient, manifesting Kaposi's disease in the course of tuberculosis, with the aim to assess this uncommon disorder and to outline this rare atypical association.

## 1. Introduction

Kaposi's Sarcoma (KS), was first described in 1872, by Moritz Kaposi, is a chronic and proliferative disorder with both a vascular and a fibroblastic component.

Four different clinical presentations can be distinguished: the classical standard one, the endemic disease, the iatrogenic form related to transplantation or to immunosuppression, and the AIDS-related epidemics [1].

Kaposi's disease is the most common malignant infection that occurs throughout VIH/AIDS affected patients [2–4].

Our medical observation relates to the case of an HIV-negative examinee having a Kaposi's Sarcoma-associated pulmonary tuberculosis.

## 2. Case Report

Our 70-year-old male patient is followed for a bacteriologically proven lung tuberculosis. One month after starting treatment, there were emergence of nodular lesions on the extremities in context of fever, and alteration of general state of health, prompted the subject to halt the antibacillary treatment.

**Figure 1:**
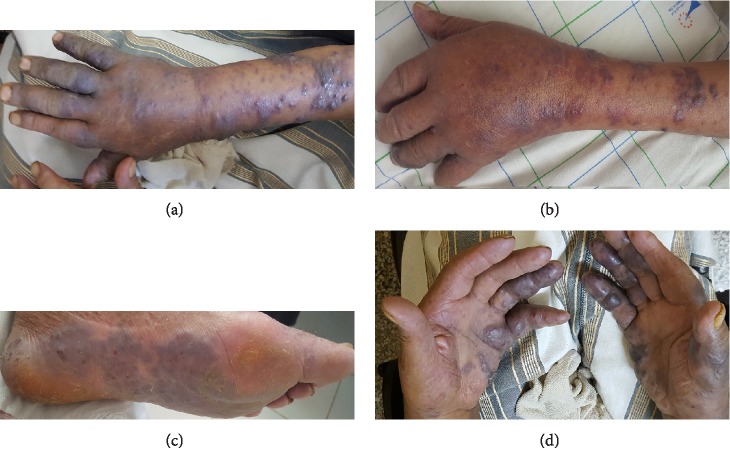
(a) Forearm cutaneous lesions. (b) Hand lesions extension. (c) Plantar cutaneous lesions. (d) Palmar cutaneous lesions.

In clinical examination on admission, cutaneous findings included sore infiltrated purplish papulonodular lesions on both the forearms (Figure 1(a)), with a rapid extension on the two hands (Figure 1(b)). Papillomatous, palmoplantar skin lesions, as well as important lymphedema of the extremities were also observed (Figures 1(c) and 1(d)). Pleuro-pulmonary examination showed no abnormality and the rest of physical assessment had no peculiarities.

Biologically, the patient had a normochromic, normocytic anemia with a hemoglobin level of 10, 5 g/dl, a white blood cell count of 8520/mm^3^ and a lymphocytopenia level of 700 per mm^3^. The C-reactive protein was at 94 mg/l and the erythrocyte sedimentation rate was at 45 mm in the 1^st^ hour. The search for Koch's bacillus (*Mycobacterium tuberculosis*) on direct examination was negative. Serological assays for HBV (viral hepatitis B), HCV (viral hepatitis C), and HIV (human immunodeficiency virus) were negative as well.

**Figure 2:**
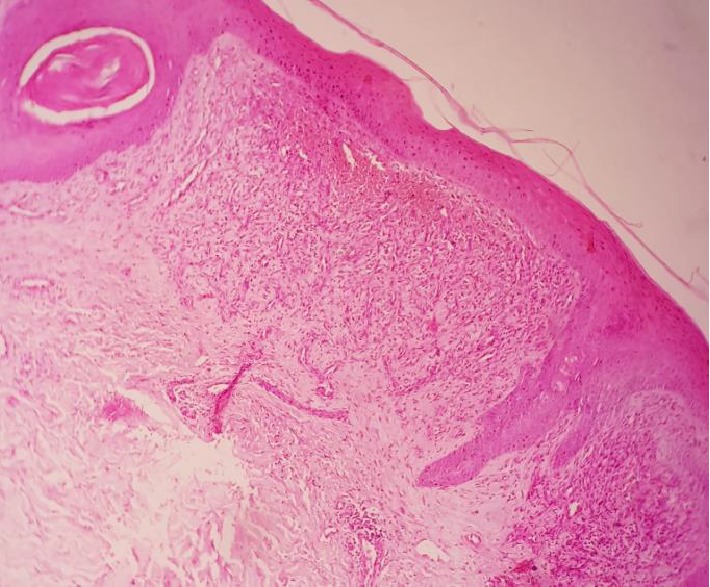
Skin biopsy results.

Cutaneous biopsy revealed proliferation of fusiform and vascular cells with extravasation of blood associated with occasional siderophages (Figure 2). Immunohistochemical studies resulted in Kaposi's Sarcoma.

The patient has been placed for two months under a four-drug anti-tuberculosis regimen made from Isoniazid (H/Inh), Rifampin (R/Rif), Pyrazinamide (Z/Pza), and Ethambutol (E/Etb), followed by a dual therapy based on Isoniazid and Rifampin. After two months, the clinical outcome was favourable with an improvement of the general condition, a decrease in the extent of skin lesions' and a partial regression of lymphedema starting from the fourth month of treatment.

## 3. Discussion

Several factors are involved in Kaposi's disease pathogenesis. In fact, we can cite genetics, HHV8 viral infection (human herpes virus 8), as well as compromised immune system following an HIV infection, recourse to immunosuppressive agents, lymphoproliferative disorders (LPDs), and far less frequently tuberculosis [4, 5].

Our case was an HIV-negative patient with both pulmonary tuberculosis and Kaposi's Sarcoma.

Wang et al. [4] described the case of a subject who presented, within five months of kidney transplantation, a tuberculous adenitis associated with extensive Kaposi's disease primarily involving mucous membranes, the digestive tract, the lungs, and the mediastinum. He benefited from anti-tuberculosis therapy that continued over the course of one year to a spectacular regression of all Kaposi's Sarcoma's lesions.

Lanjerwar [5] and Castro [6], for their part, have documented, respectively, the case of two individuals whose first was HIV-positive while the other was seronegative. Both had multi-focal tuberculosis with the lung, the liver, and the spleen involvement, together with Kaposi's Sarcoma.

Alongside our observation, Guler et al. [7] exposed a rare case of Kaposi's Sarcoma that had developed in an HIV-negative subject suffering from pulmonary tuberculosis.

Ajili [8] and Chen [9] studies concerned the emergence of Kaposi's Sarcoma on a ground of ganglionic if not cutaneous, or even miliary tuberculosis inducing immunosuppression through cellular immune deficiency [10].

The clinical course of our patient, was characterized by the improvement of the general health along with gradual decline of the cutaneous lesions, were consistent with literature data [7, 8] whereby only Chen et al. [9] deplored their examinee's death.

The onset of Kaposi's Sarcoma (KS) was explained by a cell immune deficiency caused by HIV infection. Thus, it has been shown that the proportion of KS was 7000 times higher among HIV-positive patients than among HIV-negative [11] ones and arises 300 times more in the aftermath of iatrogenic immunosuppression (attributable to a transplantation or an immunosuppressive therapy) [12, 13].

The peculiarity, of our case report lies in the fact that KS took place with the presence of no other cause of immunosuppression besides tuberculosis, which has been reported only rarely in the literature.

For that matter, the regression of KS's lesions under anti-tuberculosis drugs, in the absence of specific medical treatment such as chemotherapy, substantiates this assessment.

## 4. Conclusion

Kaposi's Sarcoma (KS) is a pathological entity, its presence along with tuberculosis in immunocompromised patients have been reported only rarely in literature. While facing a Kaposi's disease, the research of immunosuppressive factors are in order. Although tuberculosis has very rarely been reported, this affection should be sought in our country where it remains endemic.
